# Spinal Cord Injury Research in Mice: 2008 Review

**DOI:** 10.1100/tsw.2009.63

**Published:** 2009-06-12

**Authors:** Inge Steuer, Pierre A. Guertin

**Affiliations:** ^1^ Faculty of Medicine, Department of Anatomy and Physiology, Laval University, Quebec City, Quebec, Canada; ^2^ Neuroscience Unit, Laval University Medical Center (CHUL - CHUQ), Quebec City, Quebec, Canada

**Keywords:** spinal cord, lesion, transection, neuron, health degradation

## Abstract

Spinal cord injury (SCI) is an irreversible condition causing damage to myelinated fiber tracts that carry sensation and motor signals to and from the brain. SCI is also associated with gray matter damage and often life-threatening secondary complications. This mini-review aims to provide the nonspecialist reader with a comprehensive description of recent advances made in 2008 using murine models of SCI. A variety of approaches, including advanced genetics and molecular techniques, have allowed a number of key findings in the field of secondary degeneration, repair, regeneration (including insights from peripheral nerve lesion models), metabolic dysfunctions, and pharmacological neuromodulation.

## INTRODUCTION

For a long time, spinal cord injury (SCI) and paralysis were considered severe and irreversible conditions. It was believed that neurons in the central nervous system (CNS, brain and spinal cord) could absolutely not regrow once injured, unlike nerves in the peripheral nervous system (PNS). However, since the 1980s, breakthrough findings, mainly in rat models of SCI, have shown that partial regrowth is possible under some circumstances (e.g., grafting using PNS tissue) in laboratory conditions. In the late 1980s and early 1990s, some proteins associated with oligodendrocytes (cells that create myelin sheaths) were reported to prevent significantly spinal cord neuron regeneration and repair after trauma in rats. It was also found that the glial scar that forms after SCI is a significant barrier to axon regrowth.

However, a shift toward mouse models of SCI has occurred recently, mainly because of their usefulness as a genetic tool. Indeed, more is known about the genetics of mice than any other mammal, and an increasingly large number of spontaneous mutants or genetically engineered murine models are now commercially available to study SCI and other CNS indications.

Perhaps two of the fields where research in mice has led to significant advances are stem cell technology and locomotor central pattern generator (CPG) circuit genetics. For instance, synergy between immune cells and adult neural stem/progenitor cells was found, using green fluorescent protein (GFP) technology, to promote functional recovery after SCI[1]. Mice were also used to establish that netrin-1 is a critical regulator of adult spinal cord progenitor cells during development as well as after SCI[2]. A key role for glial fibrillary acidic protein (GFAP) and vimentin genes in axonal regenerative failure after SCI was revealed using double-mutant mice[3]. CM-101, an antiangiogenic molecule, was found to induce significant functional recovery in mice after SCI[4]. Pax6-knockout mice were used to show that the transcription factor Engrailed 1, expressed specifically in CPG candidate V1 interneurons, is critically involved in the control of speed[5], whereas the EphA4 receptor and its ligand EphrinB3 were found to underlie CPG candidate neurons involved in bilateral coordination during walking[6]. Insights into the pharmacological control of CPG neurons (e.g., 5-HT1A, 5-HT2A, and 5-HT7 receptors) have also been gained using wild-type and knockout spinal cord–transected mouse models (e.g., [7,8]).

As shown in this review, a great variety of topics were explored by researchers in 2008 using new mouse models. For instance, two new mouse models of SCI using either graded forceps or Allen's weight drop system were developed. Other laboratories provided new insights into mechanisms underlying secondary degeneration and scar formation associated with Beclin-1, dendritic cells, interleukin-12, neutrophil infiltration, aquaporin-4, isolectin B4, 5-lipoxygenase inhibitors, ethyl pyruvate, and prostaglandin D2. Investigations aimed at further understanding axonal regeneration using CD11b-TK(mt-30) mice or Semaphorin3C expression were also conducted. Some of the cellular mechanisms underlying central regeneration were unraveled using 2-3-cyclic nucleotide 3-phosphodiesterase promoter – enhanced green fluorescent protein (CNP-EGFP) mice or recombinant transforming growth factor (TGF)-alpha. New cell replacement approaches were assessed using genetic fate mapping or neurally modified mesenchymal stem cells. Finally, mechanisms underlying metabolic and systemic problems associated with chronic SCI, as well as alternative approaches for functional recovery based on spared neuronal network neuromodulation, have been explored.

## ANIMAL MODELS

Although a number of animal models of SCI already exist, some of the approaches developed in larger mammals may be difficult to use in mice. Therefore, a group of researchers has developed a novel crush SCI model using graded forceps for mice[9]. Three different forceps were tested with spacers of 0.25, 0.4, and 0.55 mm, to produce severe, moderate, and mild injuries, respectively. In mice subjected to a laminectomy (T5-T7 level), they found that a 15-sec spinal cord crush using one of those forceps produced proportional tissue losses and corresponding locomotor function deficits. Given the consistency of their results, the authors concluded that this approach may constitute an attractive alternative since it is easy to use, affordable, and clinically relevant[9]. Another model of SCI using an Allen's weight drop system was also developed[10]. It was reported to reflect nicely the pathological and physiological features found at different degrees of SCI. Although first described in rats, an adaptation of these injury models in mice is likely to promote further the use of mice for SCI research.

## SECONDARY DEGENERATION AND SCAR FORMATION

It is well recognized that SCI triggers a robust inflammatory response and cell death that contributes to the secondary degeneration of initially spared tissue. Kanno and colleagues[11] examined the effects induced by Beclin 1, a Bcl-2 interacting protein, known to promote autophagic cell death in various pathological conditions. Its expression in neurons, astrocytes, and oligodendrocytes was found to increase rapidly (4 h postlesion) and long lastingly (up to 21 days postlesion) at the site of injury after hemisection. This finding suggests that Beclin 1 may play a role in autophagic cell death after SCI[11].

Another approach was tested to reduce the immune-driven inflammatory response occurring after SCI. Vaccination using dendritic cells pulsed with homogenate proteins from the spinal cord (i.e., believed to contain several kinds of autoimmune antigens) was found, on systemic (i.p.) or local (site of injury) administration, to promote functional recovery after moderate SCI significantly in mice. In fact, at

84 days postinjection, treated mice displayed Basso, Beattie, and Bresnahan (BBB) scores as high as 18, whereas BBB scores around 10 were reported in control (PBS-treated) animals[12].

Along this line of evidence, administration of interleukin-12 (normally secreted by dendritic cells) into the injured site was found to increase the number of activated microglia/macrophages and dendritic cells, as well as the expression of brain-derived neurotrophic factor (BDNF) around the lesion site in SCI mice[13].

A new approach using blood cytometry was used to quantify the inflammatory response after SCI. Researchers found that CD45 (common leukocyte antigen), CD11b (complement receptor-3), Gr-1 (neutrophil/monocyte marker), and CD3 (T-cell marker) increased shortly after SCI (at 12 h). At 24 h, monocytes increased, whereas lymphocytes significantly decreased. This approach also revealed that the majority of the blood-derived infiltrate at the site of injury were neutrophils and showed that lymphopenia may contribute to secondary degeneration after trauma[14].

To further explore the mechanisms underlying inflammation and scar formation, British scientists examined the effects of eliminating aquaporin-4 (AQP4), a water-channel protein expressed in astrocytes, on scar formation after spinal cord compression. They showed that both locomotor (BBB near 5) and sensory functions were significantly increased in AQP4-deficient mice compared with control (BBB near 1.3). This corresponded to less neuronal death, less myelin vacuolation, and reduced spinal cord swelling at 2 days following a midthoracic compression in AQP4-null animals. These results suggest that AQP4 water channels in astrocytes are involved in scar formation and corresponding cell death[15].

Destruction of blood vessels at the site of injury is believed to contribute to secondary degeneration. Intravenous injection of isolectin B4 was shown in SCI mice to constitute a useful approach to assess the functional status of neovascular formation and reactive microvascular elements at the site of injury[16].

To assess the potential of anti-inflammatory drugs on reduced secondary degeneration, a group of scientists examined the effects induced by 5-lipoxygenase (5-LO) inhibitor (zileuton) and a cysteinyl-LT (Cys-LT) receptor antagonist (montelukast) in SCI mice. Mice treated with zileuton or montelukast displayed reduced spinal cord inflammation, tissue injury, neutrophil infiltration, tumor necrosis factor (TNF)-alpha, cyclooxygenase-2 (COX-2) and phosphorylated extracellular signal-regulated kinase 1/2 (pERK1/2) expression, prostaglandin E(2) (PGE[2]) and leukotriene B(4) (LTB[4]) production, apoptosis, and improved recovery of limb function over 10 days[17].

The same group of researchers also examined the effects of ethyl pyruvate (EP) in SCI mice. EP (75, 25, or 8.5 mg/kg) administered 1 and 6 h after the SCI significantly decreased spinal inflammation, tissue injury, neutrophil infiltration, nitrotyrosine formation, inducible nitric oxide synthetase (iNOS) expression, proinflammatory cytokines expression, nuclear factor kappaB activation, ERK1/2 mitogen-activated protein kinase (MAPK) phosphorylation, apoptosis, and increased dose-dependent locomotor function recovery[18].

C1q knockout mice were instrumental to demonstrate a significant role for the corresponding pathway in macrophage response, tissue sparing, and functional recovery after SCI[19].

Hsu and colleagues utilized MM-9 null mice to show clearly a determinant role for matrix metalloproteinase-9 in scar formation after SCI[20].

Lisa McKerracher's group published data showing evidence of preclinical efficacy (increased functional recovery) and safety (normally increasing body weight) in mice and rats following topical administration of BA-210, a cell-permeable fusion protein derived from C3 transferase, on the spinal cord of acutely injured animals[21].

Jakeman's group showed unsuspected data using MRL/MpJ mice. Those animals were found, paradoxically, to display impaired locomotor recovery simultaneously with increased regeneration and reduced inflammatory response[22]. They believe that this may be caused by a lost ability of the system to restore tissue integrity after insult in these mutant animals despite exceptional tissue regenerative capacity.

Using the Cre recombinase (Cre)-loxP system under regulation of the mouse GFAP promoter, a determinant role for the transcription 3 (STAT3) from astrocytes was reported in astrogliosis and scar formation regulation[23]. Indeed, conditional deletion of STAT3 (STAT3-CKO) from astrocytes in

GFAP-Cre reporter mice led to attenuated up-regulation of GFAP, failure of astrocyte hypertrophy, and pronounced disruption of astroglial scar formation after SCI.

Finally, neuroprotective effects against secondary degeneration were found with a metabolite of prostaglandin D2 (PGD2) in spinal cord–contused mice. Given daily for the first 2 weeks, PGD2 led to significant improvement of sensory and locomotor functions, reduced signs of microglia activation, and reduced demyelination, suggesting clear anti-inflammatory effects induced by PGD2 after SCI[24].

## AXONAL REGENERATION

Using a peripheral nerve lesion model, Barrette and colleagues[25] found that axonal regeneration promoted by local ganciclovir administration critically depends on CD11b+ cells. In fact, continuous delivery of ganciclovir at the sciatic nerve lesion site greatly decreased the number of granulocytes/inflammatory monocytes and macrophages in the distal stump of CD11b-TK(mt-30) mice (i.e., mice in which the HSV-thymidine kinase [TK] gene is expressed under the control of the CD11b gene promoter, so that ganciclovir would eliminate conditionally proliferating microglia and macrophages at selected time points pre- and postlesion). Axonal regeneration and locomotor function recovery were severely compromised in ganciclovir-treated CD11b-TK(mt-30), providing strong evidence that myeloid cells can support axonal regeneration and functional recovery by creating a growth-permissive milieu for injured axons[25].

Lasiene and colleagues[26] showed signs of spontaneous remyelination in SCI (contusion) mice. Indeed, spared rubrospinal tract (RST) axons of passage traced with actively transported dextrans were fully remyelinated caudally to the lesion at 12 weeks postinjury in mice. However, using mathematical models and internode and paranode protein distribution data, they assessed that remyelinated tracts displayed a 21% decrease in conduction velocity attributable to shortened internodes[26].

A group of researchers used two adult mouse models to evaluate the neuronal expression of Semaphorin3C (Sema3C is an axonal guidance molecule better known for its role in endothelial cell guidance) in facial motoneurons (FMNs) and rubrospinal neurons (RSNs) following axonal injury[27]. With *in situ* hybridization (ISH), they reported a transient up-regulation in Sema3C mRNA expression in injured FMNs as well as in injured RSNs, although Sema3C expression was not detected prior to injury only in RSNs, suggesting, in turn, that Sema3C is unlikely to influence axonal regeneration directly[27].

## CELLULAR MECHANISMS OF REGENERATION

Given that the chondroitin sulfate proteoglycan (CSPG) NG2 is a marker for oligodendrocyte progenitor cells (OPCs) during development, some investigators were interested in studying NG2(+) cells after SCI in CNP-EGFP mice. After SCI, EGFP(+)/NG2(+) cell proliferation in residual white matter peaked at 3 days postinjury rostral to the epicenter, whereas EGFP(-)/NG2(+) cell proliferation peaked at 7 days postinjury at the epicenter. These results suggest that NG2(+) cells (i.e., at least those that normally function as OPCs) respond after SCI as they do normally during embryonic developmental oligodendrogenesis[28].

Along the same idea, scientists from the Weizmann Institute (Israel) reported that CSPG may play a key role during the acute recovery stage after SCI in mice[29]. They showed data suggesting that inhibition of CSPG synthesis immediately after injury impaired functional motor recovery and increased tissue loss. However, using chimeric mice, they also found that delayed inhibition of CSPG production improved functional recovery, suggesting the existence of a time-dependent role for CSPG-promoting regeneration and repair in the acute phase, but in increased tissue loss at later stages post-SCI[29].

Although astrocytes are well known to contribute to glial scar formation, there is also evidence to suggest a role in neuroprotection. To further investigate this avenue, recombinant TGF-alpha (a mitogenic growth factor that is active on astrocytes) was administered (i.t.) during 2 weeks in moderately SCI

mice[30]. TGF-alpha infusion did not affect locomotor recovery, but promoted axon growth. They also found a marked increase in axons expressing neurofilament and growth associated protein 43 (GAP-43) immunoreactivity, suggesting that on appropriate stimulation, endogenous astrocytes can promote axon growth after SCI[30].

A transgenic yellow fluorescent protein (YFP-H) mouse model was used to reveal novel neuroprotective mechanisms (ERK1-mediated) underlying chondroitinase ABC–mediated repair post-SCI[31].

Spinal cord–hemisected mice injected bilaterally with function-blocking Ryk antibodies into the dorsal spinal cord prevented the retraction of corticospinal tract (CST) axons, or promoted their regrowth around and beyond the injury site, suggesting that repulsive Wnt signaling (i.e., well known also for its role in embryogenesis and cancer) may be a cause of CST axon retraction and inhibits axon sprouting post-SCI[32].

## CELL REPLACEMENT AND SCI REPAIR

Meletis and colleagues[33] studied endogenous neural stem cells in the murine spinal cord. Using genetic fate mapping, they showed that most *in vitro* neural stem cell potential in the adult spinal cord resides within the population of ependymal cells lining the central canal. After SCI, these cells are recruited to produce not only scar-forming glial cells, but also oligodendrocytes, suggesting that modulating the fate of ependymal progeny after SCI may offer an alternative to cell transplantation for cell replacement therapies after SCI.

Another team of researchers reported a method to neurally modify mesenchymal stem cells (MSCs from the bone marrow)[34]. They recently reported that several mature neural markers, such as B3T, GFAP, MAP-2 (mitogen-activated protein 2 kinase), NF-200 (neurofilament 200 kDa), and NeuN (neuronal-specific nuclear protein), were found in their *in vitro* neurally modified MSCs. They found also that such cells release nerve growth factor (NGF) and BDNF. Grafting into an intact spinal cord (rats), these cells from murine models were found to survive and express neural markers, suggesting that neurally modified MSCs could constitute a novel approach for cell replacement after SCI[34].

Bretzner and colleagues[35] actually reported unsuspected side effects following a combined treatment with olfactory ensheathing cells (from GFP-mice) and BDNF. Indeed, while both treatments used individually have been shown to promote repair and functional recovery post-SCI several times, when combined, these approaches increasingly impaired motor performance in treated SCI rats.

In chronically hemisected SCI mice, propriospinal reorganization and sprouting sublesionally was found to be critically important for functional recovery induced by regular treadmill training in wild-type mice[36].

Along the same line of research, the capability of corticospinal tracts to sprout and regenerate after SCI failed to be shown following NEP1-40 treatment (Nogo extracellular protein residue 1-40 antagonist) in partially SCI mice (independent replication of Li and Strittmatter's study)[37].

## SYSTEMIC, METABOLIC AND OTHER NON-LOCOMOTOR COMPLICATIONS

A large variety of systemic, metabolic, and nonlocomotor-related functions are either lost or altered in chronic SCI subjects (for a recent review, see Ung et al.[38]). For instance, adult mice were shown to undergo a rapid and extensive loss of bone tissue following a complete transection of the spinal cord. Picard and colleagues reported, indeed, a 22, 10, and 14% decrease in femoral bone volume, trabecular thickness, and trabecular number, respectively[39].

Sexual dysfunction is also typically found after SCI. Rezaian and colleagues[40] studied the role of sperm motility in CatSper genes known to encode for a unique Ca(2+) channel in the testes. They found a significant down-regulation of CatSpers 1 and 2 several weeks after SCI in mice (but not in controls), suggesting that some of the problems encountered with sperm viability and motility after SCI may be associated with the down-regulation of these genes.

Although frequently found after SCI, chronic pain is incompletely understood. Using a novel line of double glutamate transporter (GLT-1 and GLAST) transgenic GLT-1-enhanced GFP (eGFP)/GLAST-Discosoma Red (DsRed) promoter mice, Tawfik and colleagues[41] showed that propentofylline (atypical methylxanthine)-induced astrocyte modulation may alter glial glutamate promoter activation following peripheral nerve transection. Adult mice that received propentofylline i.p. starting 1 h prior to L5-nerve transection and then daily for 12 days exhibited antiallodynic properties, suppressed astrocytic activation, and reversed glutamate transporter reduction.

Another drug believed to reduce chronic pain was tested in nerve-ligated mice. Milnacipran administered systemically, i.t., and i.c.v. increased withdrawal threshold and withdrawal latency in nerve-ligated mice, whereas local injection in the dorsal horn had no effect[42]. The effect of milnacipran was lost after noradrenergic denervation (6-hydroxydopamine-treated), but not after serotonergic denervation (5,7-dihydroxytryptamine–treated), suggesting that the antiallodynic and antihyperalgesic effects of milnacipran on neuropathic pain induced by peripheral nerve ligation were mediated through both supraspinal and dopaminergic spinal mechanisms[42].

Finally, a role in chronic pain for the transient receptor potential vanilloid 1 (TRPV1) was also shown using TRPV1 knockout mice[43], and daily administered purinergic antagonists (PPADS) were shown to reduce neuropathic pain, NO/NOS, and interleukin-1beta expression in peripheral nerve-injured mice[44].

## FUNCTIONAL RECOVERY ASSOCIATED WITH DISTAL NEURONAL NETWORK

Most strategies currently developed by scientists to promote functional recovery after SCI are based on approaches aimed to repair the spinal cord. However, an increasingly considered approach is based on activating and modulating the activity of spared neuronal networks distally located from the site of injury. Lapointe and colleagues[45] studied the effects of adrenergic agonists on CPG (i.e., in the lumbar cord) reactivation in low-thoracic spinal cord–transected (Tx) mice. In contrast with results previously reported in cats, clonidine (alpha-2 agonist) was found not to induce CPG-mediated hindlimb locomotor movements in Tx mice[45] with or without simultaneous sensory stimulation (tail pinching)[46]. They also reported that clonidine acutely suppressed, through imidazoline receptor activation, the effects of CPG-activating compounds induced by 8-OH-DPAT, a 5-HT1A/7 receptor agonist[45].

In contrast, the partial pro-CPG-activating effect of 8-OH-DPAT was found to be synergistically enhanced following coadministration with dopamine receptor agonists in Tx mice. Indeed, Lapointe and Guertin[47] showed that full CPG-activating effects, including large amplitude stepping, weight bearing, and plantar foot placement, were induced in untrained, nonassisted, and nonsensory-stimulated Tx mice following administration (s.c. or i.p.) of 8-OH-DPAT and SKF-81297 or, to some extent, apomorphine. Finally, the same group of researchers also recently reported that among the 5-HT2 receptor subtypes, only the 5-HT2A receptor was specifically associated with partial CPG-activating effects in Tx mice[48].

## CONCLUSION

All and all, the use of murine models of SCI has drastically increased in the last decade and, again, significant findings were made in 2008. Insights were provided into mechanisms underlying secondary degeneration and scar formation associated with Beclin-1, dendritic cells, interleukin-12, neutrophil infiltration, AQP4, isolectin B4, 5-LO inhibitors, EP, and PGD2. Some of the mechanisms underlying central regeneration were unraveled using CNP-EGFP mice or recombinant TGF-alpha. New cell replacement approaches were assessed using genetic fate mapping or neurally modified MSCs. Mechanisms underlying metabolic and systemic problems associated with chronic SCI, as well as alternative approaches for functional recovery based on spared neuronal network neuromodulation, have been explored.

New murine models and molecular tools will likely continue to help scientists to understand further the complex pathological consequences of acute and chronic SCI. Given the new direction taken by the Obama administration in 2008 regarding stem cell research, we may expect more funding and breakthroughs in transplantation and spinal cord repair. It will become increasingly important to develop further multidisciplinary approaches to bridge the gap between genetically, electrophysiologically, pharmacologically, and behaviorally characterized CPG-candidate neurons. Although the field of secondary health complications after SCI has not previously attracted as many researchers as spinal cord repair and regeneration, it is critically important to increase largely drug discovery and development efforts for the development of innovative therapeutic approach against life-threatening secondary complications after SCI.
